# Orangutans (*Pongo pygmaeus*) recognize their own past actions

**DOI:** 10.1098/rsos.181497

**Published:** 2018-12-12

**Authors:** Yuki Hanazuka, Mika Shimizu, Hidemasa Takaoka, Akira Midorikawa

**Affiliations:** 1 Institute of Cultural Science, Chuo University, Hachioji, Tokyo 192-0393, Japan; 2 Faculty of Letters, Chuo University, Hachioji, Tokyo 192-0393, Japan; 3 Tama Zoological Park, Hino, Tokyo 191-0042, Japan; 4 Ueno Zoological Gardens, Taito-ku, Tokyo 110-0007, Japan

**Keywords:** delayed self-recognition, orangutan, preferential-looking paradigm

## Abstract

The ability to recognize oneself in a mirror is known as self-recognition, whereas delayed self-recognition is the ability to recognize the relationship between current self and past actions. While 3-year-old human children have self-recognition without the ability for delayed self-recognition, 4-year-old human children demonstrate the capability for both. Chimpanzees, the most closely related species to humans, have displayed the ability for delayed self-recognition. However, little is known about whether this ability is shared among all hominid species. In this study, we examined whether orangutans, the most distantly related species to humans within the hominid group, could recognize their own past actions using the preferential-looking paradigm. Our results demonstrated that orangutans were able to discriminate between a delayed video of themselves presented after a 2-s delay and a recorded video of the day prior. This suggests that orangutans have the ability to relate their own past actions to current actions, although we found no evidence of self-directed behaviour. We believe these findings will contribute to our growing understanding of hominid self-recognition.

## 1. Introduction

Self-recognition is the ability to recognize the contingency between one's own actions and the mirror image. This was previously investigated with the mirror self-recognition (MSR) task, in which the subject's face is marked with a spot and then the subject is presented with a mirror. When subjects possess the ability for self-recognition, they will inspect the spot on their face upon seeing their reflection [1–3]. By about 18–24 months of age, human infants will use a mirror (or live video) feedback to check a mark placed covertly on their face [4]. These studies demonstrate that even young children are capable of understanding the correlation between one's own actions and the actions observed in the mirror.

Older children possess a more mature concept of self-through-time, which serves to connect the present and past selves. Povinelli suggested that there are two types of self [5]: ‘present self’, which is examined through the MSR task; and ‘proper’ or temporally extended self that combines one's past actions with current actions. The ‘proper’ self is considered a higher-order representation of self. It is known that 3-year-old infants possess self-recognition in a mirror but are not able to recognize their own actions on video with a 2-s delay [6]. This indicates that temporal contingency is required for 3-year-old infants to understand the correlation between their self-action and self-image. Furthermore, Povinelli used the delayed self-recognition task with photographs or video feedback and investigated whether children could understand the temporal–causal relation between their ‘past self’ and ‘present self’ [5,7]. The data indicate that young children do not gain a sense of the ‘proper self’ until the fourth year of life [5,8].

From the viewpoint of comparative cognitive studies, there have been many investigations on self-recognition ability in nonhuman animals. In a landmark study, Gallup demonstrated that chimpanzees have the capacity for self-recognition using mirrors [9]. Gallup anaesthetized a chimpanzee and covertly marked its face with rouge. After the chimpanzee awoke, a mirror was introduced. The chimpanzee used the mirror to inspect parts of its body and did not demonstrate any social behaviours towards its own reflection. This experiment was the first to demonstrate with visible, behavioural evidence that nonhuman animals have the ability for self-recognition. Since the Gallup's [9] MSR was introduced, other researchers have used it to study self-recognition in a variety of species including gorillas [10,11], bonobos [12], orangutans [10,13], elephants [14] and dolphins [15]. These studies found that only the great apes, dolphins and elephants could pass the MSR test. These studies also suggested the presence of self-concept, although the results in gorillas remain controversial.

Other animal species may also have the ability for self-recognition, but previous research has primarily focused on the animal's reaction to the mirror. Few studies have examined whether nonhuman animals possess a temporally extended sense of self. For example, a recent study demonstrated that chimpanzees could recognize and react to a past self-action after a delay of 4 s. Several circular stickers were placed on the face and head of chimpanzees, who were then presented videos after 0- to 4-s delays [16]. Three of five chimpanzees removed the stickers after the 4-s delay, suggesting that chimpanzees could recognize a past self-action after a 4-s delay. This study indicates that great apes can also identify delayed self-images, although it has not yet been demonstrated whether they have the ability for delayed self-recognition.

This study examined whether orangutans, a great ape distantly related to humans and chimpanzees, can discriminate between a delayed video of themselves and a recorded one with a temporal delay and exhibit implicit recognition. We conducted two experiments regarding self-recognition. In experiment 1, as the preliminary study, we examined the reactions of orangutans to live self-videos and recorded videos on two monitors to confirm whether the orangutans could distinguish between synchronized and non-synchronized self-actions with the preferential-looking method, i.e. the paradigm of simultaneously presenting two images. We assessed whether the preferential-looking method is valid in the investigation of orangutan self-recognition. If the experiment only presented delayed and recorded self-video to orangutans and our results found that orangutans did not possess the ability for delayed self-recognition, we would not be able to differentiate whether it was truly an inability to recognize delayed self-video, or if the problem was based on the experimental design and we were not able to properly examine the delayed self-recognition of orangutans. We predicted that orangutans would prefer to watch a real-time video of themselves, rather than a recorded one in experiment 1. In experiment 2, we examined whether orangutans are able to discriminate between videos of themselves after a 2-s delay (i.e. one of the same temporal delay conditions used by Hirata *et al.* [16]) from recorded ones. We predicted that orangutans will spend more time looking at the delayed video of themselves in experiment 2 if they are able to relate their past self-actions to current ones. These data could be essential for understanding the evolutionary origins of information integration with respect to past occurrences of the self with an individual's current self-concept.

## 2. Material and methods

### 2.1. Participants

Three Bornean orangutans housed at the Tama Zoological Park in Tokyo, Japan, were studied: two adult females (Gypsy, approximately 58 years old, and 48-year-old Julie) and one adult male (28-year-old Borneo). Gypsy was born in the wild and has lived at the Tama Zoological Park for over 50 years. Julie was born and raised in Tama Zoological Park, and Borneo was born in Singapore Zoo and moved to the Tama Zoological Park in 1998. Gypsy and Julie were housed socially and had direct and visual contact with other orangutans. Borneo also had the opportunity to see other individuals but was housed separately. All individuals were fed fruit and vegetables, and water was available ad libitum. These orangutans have previously participated in other cognitive experiments [17].

### 2.2. Apparatus

The orangutans were tested in their resting room (1.8 m wide × 2.6 m deep × 2 m high), which is surrounded by a mesh fence (each hole in the mesh measures 5 × 5 cm). The stimuli were presented using two 17-inch liquid crystal monitors (LCDA173KB; I-O DATA, Japan) positioned diagonally to each other (150° angle) and controlled by a laptop computer (SL500 2746-7DJ ThinkPad; Lenovo, Hong Kong). A Web camera (QCAM-200SX Logicool; Logitech, USA) was placed on each monitor to record and display the face and body of the orangutans. The distance between the participant and the monitors was approximately 60 cm. A video camera (HDR-PJ790; Sony, Japan) was placed on a tripod between the monitors and a laptop was used to record the gaze of each orangutan. To create real-time and delayed self-videos, we used a device (Sports Mirror; New Forestar Co. Ltd, Japan) that can display images of individuals after a delay of 0–20 s in the same left–right orientation on the monitor as seen in a mirror. We used GOM Player (Gretech Corporation., South Korea) to display the recorded video. All stimuli were presented in full-screen format.

### 2.3. Stimuli

We prepared three types of stimuli that differed in temporal properties: a real-time self-video presented with no temporal delay, a delayed self-video presented after a 2-s delay and a self-video that was recorded the day before the experiment. During the experiment, participants were simultaneously presented either a real-time self-video and a recorded self-video, or a 2-s delayed self-video and a recorded self-video.

### 2.4. Procedure

Experiment 1 started when an orangutan spontaneously moved in front of the apparatus. The experimenter initiated the experiment as soon as the orangutan began paying attention to the monitor. All orangutans were tested under the same experimental conditions, which comprised a 5-min recording phase and a 5-min test phase. These two phases were separated by 1 day to avoid habituation of the orangutans to the stimuli. During the recording phase, the Web camera on the left monitor recorded the actions of the orangutan for 5 min. One day later, the Web camera on the right monitor was used to record the actions of the orangutan, also for 5 min. During the recording phase, a real-time self-video was presented on the monitor to attract the orangutan's attention to the monitor (we confirmed that orangutans actually paid attention to the monitor with this procedure). These videos constituted the recorded self-videos. During the test phase, one monitor displayed a real-time self-video, and the other a recorded self-video, to each orangutan. A session ended when the experimenter switched off the monitors and two sessions were conducted per month to prevent the orangutans from habituating to the stimuli. The position of the stimuli (left or right side) was counterbalanced between sessions. Orangutans were not subject to any restrictions on their behaviour during the recording and test phases. The total viewing time for each monitor was recorded using the video camera, which was positioned on a tripod behind the two monitors. Experiment 2 was the same as experiment 1, except that the 2-s delayed self-video was used instead of the real-time self-video. To avoid interfering with the behaviours of the orangutans being assessed, the experimenter and other individuals remained out of view during the experiment.

### 2.5. Data analysis

Each recorded video of orangutan looking behaviour was divided into 10-s parts, each of which constituted a trial. There were two sessions (300 s per session) in each experiment, and each session was divided into 30 segments (10 s each). This method, which divides sessions into several segments, previously confirmed the validity for analysing the perceptive abilities of human newborns [18] and could be useful in analysing the perceptive abilities of individuals with short attention spans. In our preliminary experiments, we observed that the attention span of an adult orangutan was as short as that of a newborn baby. We also determined that the frequency of an orangutan's looking at either stimulus for 1 s was 99% (107/108 of trials in experiment 1) and 91% (83/91 of trials in experiment 2). Therefore, we adopted the method that the sessions were divided into several segments in the current study. Any trial in which an orangutan did not look at both stimuli for more than 1 s was excluded from the analysis. Each orangutan's looking time was analysed separately. We measured the total time looking at each side on two monitors. One of the authors (Y.H.) classified gaze direction into three categories: looking at the left monitor, looking at the right monitor and not looking at either monitor. To ensure the reliability of this assessment, a second scorer with no knowledge of the stimuli presented on the display (as was also the case for the first scorer) analysed 20% of all trials. According to Cohen's kappa, the level of inter-rater reliability in this study was relatively high (*κ* = 0.88). There were 53 trials available for analysis for Gypsy, 34 for Julie and 21 for Borneo in experiment 1, and 9, 54 and *2*8, respectively, in experiment 2. This study used the row data of looking time, rather than reporting the percentage of time subjects looked at the stimulus because a recent study suggested that data transformation could have negative consequences and that raw data should be used to maintain the ratio scale [19]. Moreover, we counted self-directed or other-directed behaviours (i.e. viewing or touching the mark on unseen body parts without a mirror or monitor [9]). However, none of these behaviours were found; therefore, we did not calculate the degree of inter-rater reliability for these behaviours.

To compare the looking time of the orangutans between real-time self-videos and recorded self-videos in experiment 1, and between the delayed self-videos and recorded self-videos in experiment 2, we ran a generalized linear mixed model (GLMM), which enabled us to estimate the parameters for both fixed effects (the type of stimuli and the number of trials) and random effects (the variance between unobservable effects resulting from individual differences). GLMM can also accommodate the non-normal distributions typical of behavioural data without directly transforming the raw data, thus maintaining the ratio scale. Given that the responsible variable was the looking time, we set a Poisson distribution. The statistical model fittings were performed using R v. 3.5.1 [20] equipped with the glmmML package [21]. A *p*-value of 0.005 was considered statistically significant, based on previous recommendations [22].

## 3. Results

To test the ability of orangutans to integrate temporal information, we devised two experiments based on the preferential-looking paradigm. In experiment 1, three orangutans were given the opportunity to view real-time videos of themselves presented with no temporal delay and similar videos of themselves recorded the day before the experiment. We monitored the looking times of the orangutans to ascertain whether they could distinguish between synchronized and non-synchronized self-actions. In experiment 2, we used self-videos presented after a 2-s delay instead of real-time videos.

**Figure 1.**
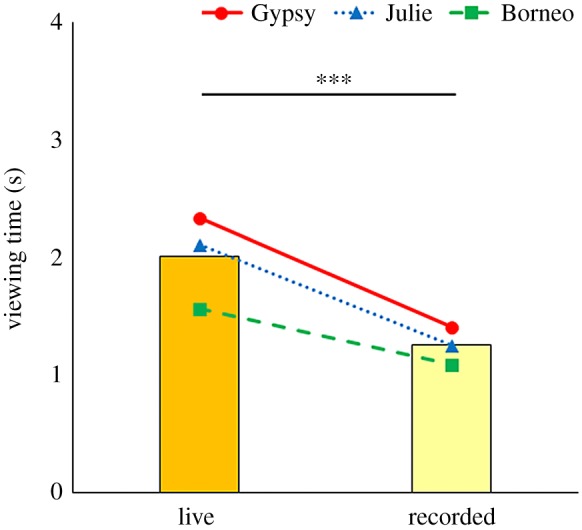
Comparison of mean looking times for the real-time and recorded self-videos. Bars represent the mean looking times in all individuals and points show each individual's mean looking times (s). ***p < 0.0001.

**Figure 2.**
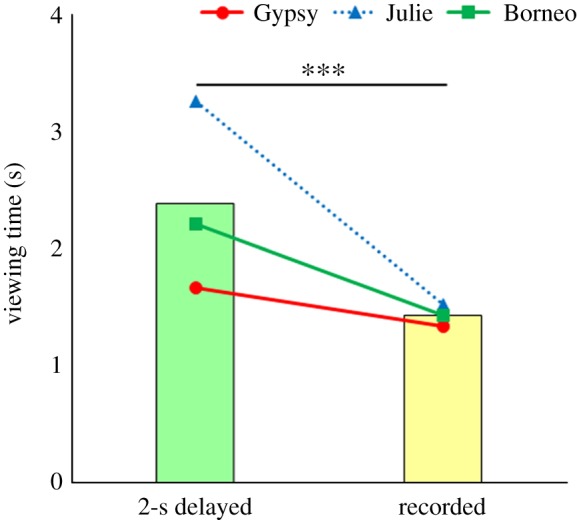
Comparison of mean looking times for the 2-s delayed and recorded self-videos. Bars represent the mean looking times in all individuals and points show each individual's mean looking times (s). ***p < 0.0001.

Figure 1 shows the results of experiment 1. The stimulus type had a significant effect on looking time (glmm: coeff. = 0.484, *z* = 4.530, *p* < 0.0001). We found no significant effect on the number of trials (glmm: coeff. = −0.011, *z* = −1.293, *p* = 0.19) in experiment 1, suggesting that orangutans spent more time looking at the real-time video of themselves than looking at the recorded one. Figure 2 shows the results of experiment 2. The stimulus type had a significant effect on looking time (coeff. = 0.636, *z* = 5.948, *p* < 0.0001), while the number of trials was not significant (coeff. = −0.016, *z* = −2.010, *p* = 0.04) in experiment 2. This suggests that orangutans spent more time looking at the delayed video of themselves than looking at the recorded one, although the number of trials might be significant when the significance level was *p* = 0.05. No testing behaviour was observed in either experiment.

## 4. Discussion

Experiment 1 confirmed that orangutans could discriminate between real-time and recorded videos of themselves, in turn suggesting that they can relate self-generated actions to contingent visual feedback. These results are consistent with previous studies in chimpanzees [23], although testing behaviours—strong evidence of self-recognition abilities—were not demonstrated. The previous study revealed that 8 of 10 chimpanzees, which are considered to possess self-recognition, did not exhibit self-directed behaviours to live self-images on television monitors [24]. Furthermore, elderly chimpanzees (16–32 years) showed fewer self-directed behaviours in the MSR task compared with younger chimpanzees (8–15 years) [25], although this does not mean that elderly chimpanzees lack self-recognition. The other study demonstrated that adolescent chimpanzees did not show self-recognition, although they displayed the ability to recognize themselves 8 years prior. Taken together, these findings suggest that elderly individuals might exhibit fewer self-directed behaviours due to their age [26]. Despite the differences between chimpanzees and orangutans, the reason why the orangutans (28–58 years) in this study exhibited no self-directed behaviour might be due to advanced age. Another study demonstrated that an adult female orangutan showed a visual preference for real-time self-images reflected by a mirror [13]. In experiment 1, we used real-time video images, which are temporally and spatially equivalent to a mirror, as analogues of mirror image to demonstrate orangutan preference for real-time images over recorded images. Thus, the results of experiment 1 described herein supported with the results of the previous study showing that orangutans can relate self-generated actions to contingent visual feedback because orangutans in both studies preferentially looked at real-time self-image, although none of the orangutans in our experiment showed any self-directed behaviours. Therefore, we believe that our paradigm captured discrimination by orangutans between real-time and recorded videos of themselves, suggesting that they can relate current kinesthetic information and external visual effects, which could be one of the abilities for self-recognition.

In experiment 2, the orangutans spent more time looking at the delayed video of themselves than at the recorded one, suggesting that orangutans possess the ability to relate past and current self-actions. Although the differences in looking time between the delayed and recorded self-videos could be a weak indicator for self-recognition, stronger evidence was needed to demonstrate delayed self-recognition. Our results suggest the possibility that orangutans could potentially have the ability to recognize the relationship between past and current self-action. In the previous study, 3-year-old children who showed self-recognition with live and 1-s delayed feedback could not identify themselves when the video had a delay of longer than 2 s [6]. These results imply that temporal causality based on immediate feedback is necessary in 3-year-olds in order to understand the relationship between their past and current self-actions. It is not until 4 years of age that children acquire the understanding of contingency between self-action and the corresponding visual with a 2-s delay. Orangutans discriminated delayed video of themselves from recorded videos, suggesting that they could recognize the relationship between past kinesthetic information and external visual effects, one of the abilities for delayed self-recognition equivalent to that of a 4-year-old child. Our results, combined with those of previous studies, suggest that the ability to relate past kinesthetic information and external visual effects for delayed self-recognition is one that we share with the great apes.

The limitations of this study included its small sample size, a lack of variation in sex and age and the total number of sessions. However, it should be noted that despite the small sample size, these results do reveal the depth of capability of the *Pongo* species [27]. The issue regarding the total number of sessions might be solved by conducting experiments over a longer time to prevent the orangutans from habituating to the stimuli. Further experiments are also needed to show that the viewing preference was truly due to self-recognition. Another possibility is that some other aspect of the videos, such as the orangutans moving around more during real-time viewing or producing more salient stimuli, increased the viewing preference of the orangutans. To exclude these possibilities, we need to perform experiments that record real-time viewing by an orangutan, and then later show the real-time and previously recorded videos to other individuals. If the viewing preference is actually due to self-recognition, then orangutans should show increased viewing time only to their own videos. Further research should investigate the ability of orangutans to detect differences between live self-video and delayed self-video, which would be considered a much higher temporal dimension of self-recognition because both stimuli are relevant to the current self's own actions. This condition was important in further understanding the temporal dimensions of self-recognition in orangutans.

Finally, future comparative cognitive studies should examine animals' self-recognition ability using experiments that assess temporal extensions in self-recognition. Although some researchers have argued that the ability to conceive of the past self is restricted to humans [28], the findings of our study suggest that even more distantly related orangutans can detect differences between a delayed video of themselves and a recorded video, implying the ability to relate past kinesthetic information and external visual effects, one of the indicators of delayed self-recognition These findings could have implications for the understanding of self-recognition in orangutans and the evolutionary development of self-recognition in hominids. Together, these experimental data provide suggestions that great apes can recognize their own past actions.

## Data accessibility

Data used in this study are available on Dryad Digital Repository: http://dx.doi.org/10.5061/dryad.b516p99 [29].
